# The effect of spinal curvature on the photogrammetric assessment on static balance in elderly women

**DOI:** 10.1186/1471-2474-15-186

**Published:** 2014-05-29

**Authors:** Justyna Drzał-Grabiec, Maciej Rachwał, Justyna Podgórska-Bednarz, Justyna Rykała, Sławomir Snela, Aleksandra Truszczyńska, Zbigniew Trzaskoma

**Affiliations:** 1 Institute of Physiotherapy, University of Rzeszów, Warszawska 26a, Rzeszów 35-205, Poland; 2 Faculty of Rehabilitation, Józef Piłsudski University of Physical Education in Warsaw, Marymoncka 34, Warsaw 00-968, Poland

## Abstract

**Background:**

Involutional changes to the body in elderly patients affect the shape of the spine and the activity of postural muscles. The purpose of this study was to assess the influence of age-related changes in spinal curvature on postural balance in elderly women.

**Methods:**

The study population consisted of 90 women, with a mean age of 70 ± 8.01 years. Static balance assessments were conducted on a tensometric platform, and posturographic assessments of body posture were performed using a photogrammetric method based on the Projection Moiré method.

**Results:**

The results obtained were analysed using the Spearman’s rank correlation coefficient test. We found a statistically significant correlation between body posture and the quality of the balance system response based on the corrective function of the visual system. The shape of the spinal curvature influenced postural stability, as measured by static posturography. Improvement in the quality of the balance system response depended on corrective information from the visual system and proprioceptive information from the paraspinal muscles.

**Conclusions:**

The sensitivity of the balance system to the change of centre of pressure location was influenced by the direction of the change in rotation of the shoulder girdle and spine. Development of spinal curvature in the sagittal plane and maintenance of symmetry in the coronal and transverse planes are essential for correct balance control, which in turn is essential for the development of a properly proportioned locomotor system.

## Background

The spine has protective, supportive and locomotive functions in the body. Spinal mobility is ensured by muscles whose initial tone is related to the shape of the spinal curvature [1-4]. Disorders which alter the shape of the spinal curvature may have a unidimensional character, meaning that curvatures may become either deepened or flattened. The pathology of muscle tone change is related to the change of the position of insertion location of a given muscle. Panjabi and subsequently Izzo used the tri-element model to explain the mutual biomechanical interdependencies. A dysfunction or pathology of one of the elements distorts the functioning of the remaining elements, and leads to insufficiency and consequent compensatory changes. Within this system is the nervous system, responsible for the steering, reception and transmission of stimuli, the spinal column, which is the passive part of the system and the spinal muscles which are the active part of the system responsible for movement and joint stability [2,5].

Involutional changes that affect the body as a result of ageing are related to the internal organs, nervous system and locomotor system. These changes constitute part of a natural process, although this process may become intensified by an incorrect lifestyle [6-9]. An example of a related characteristic feature is the sharpening of spinal curvatures in elderly people, particularly deepening of the thoracic kyphosis. Boyle et al. performed post-mortems in 113 women and 59 men of different ages. They conducted radiological assessments of the impact of age on changes to the spinal curvatures. They found thoracic kyphosis deepening in subjects of both sexes, with the kyphosis peak on the T6 vertebra. The cervical lordosis also flattened with age, especially in men, with the lordosis peak moving towards the head [10].

Furthermore, the shape and stability of individual parts of the spine are highly correlated with each another. Gardocki et al. conducted a study to define the correlation of changes between the lumbar lordosis, pelvis rotation and spinal stability in the sagittal plane. Compensation in the lumbar region has a considerable impact on the treatment of spinal disorders, the prevention and treatment of dorsum planum, and degenerative spine disorders as well as intervertebral disc protrusion [11]. Other authors have discussed the correlation of back pain to changes in response quality of the balance system, and have concluded that pain significantly worsens the condition of the balance system. This results from pain causing biomechanical changes within overburdened muscles, leading to the incorrect postural patterns [12-14].

An assessment of correlation between spinal curvatures and balance system response quality is valid, as disorders of muscle functioning result from the high sensitivity of muscles to changes in spinal curvature. This is especially true of the deep muscles of the dorsum, and the multifidus, rotatores, and intertransversarii muscles that are sensitive to the stretching reflex, which plays a role in the process of regaining balance.

Spinal curvature is an essential criterion to consider when assessing the condition of the locomotor system [15]. Spinal curvature disorders affect the dynamics and statics of the body. Effects on body statics are easy to measure through postural stability assessment using functional tests such as the Star Excursion Balance Test (SEBT), or tests on tensometric platforms which quantify balance reactions under both static and dynamic conditions [16-20].

While body posture may be assessed in several ways, the photogrammetric method is increasingly applied due to its non-invasive, repeatable and reliable nature. The method allows the assessment of several dozen parameters that characterise body posture in the coronal, sagittal and transverse planes. When correlated with parameters describing the postural stability of the patient, this allows correlations between postural stability and the shape of the spine to be explained and conclusions to be drawn that may facilitate the rehabilitation process of elderly patients.

In this study, we assessed the correlation between parameters describing static balance, as quantified during free stance on both feet with open or closed eyes, and parameters describing spinal curvatures in the sagittal, coronal and transverse planes.

## Methods

### Participants

The study population consisted of 90 women aged 60–92 years, with a mean age of 70 ± 8.01 years. The mean body mass was 72.3 ± 14.76 kg. Written informed consent for participation in the study was obtained from participants. The study design was approved by the Bioethics Committee of the University of Rzeszów. The study was conducted according to the principles outlined in the Declaration of Helsinki.

### Procedures

The test consisted of dynamic balance measurements performed in two 30-second trials, one with eyes open and one with eyes closed. Quantification was carried out using a two-plated tensometric CQstab platform. Prior to the static balance test, the subjects were asked to stand in a rectangle outlined on the floor in front of the device. The quantification was carried out in the habitual posture, with the subject standing on both feet. First, the 30-second test with eyes open was performed, followed by a 5-second break. The 30-second test was subsequently repeated with eyes closed. The device automatically calculated the Romberg quotient during the assessment. This expresses the quotient of the parameter values measured in the test with eyes open and the parameter values measured in the test with eyes closed.

The analysis employed a photogrammetric method based on the phenomenon of the projection chamber and Moiré projection. The equipment for computer evaluation of body posture employs a photogrammetry method that involves anthropometric calculations based on a photograph of the investigated surface. The device “displays” lines of strictly defined parameters on the patient's back and makes it possible to obtain a spatial image. These lines reach the patient's back at a specific angle and are distorted depending on the distance of a given point from the device. Line image distortions are recorded by a computer using numerical algorithms to convert them into a contour map of the surface. The physical basis of this method is called the Moiré phenomenon in optics. The room in which the tests were conducted was darkened. Each subject was asked to remove their clothes from the waist up and to stand in a marked location 2.6 meters away with their back to the camera on the device. Before measurements were taken, the following anthropometric points were marked on the back of each subject: spinous processes from C7 to L5, lordosis depth point, the kyphosis peak, the lower angle of the scapula, acromion, spina iliaca anterior posterior, and the thoracolumbar junction. After determining the anthropometric points, a photogrammetric image was taken [21,22].

**Figure 1:**
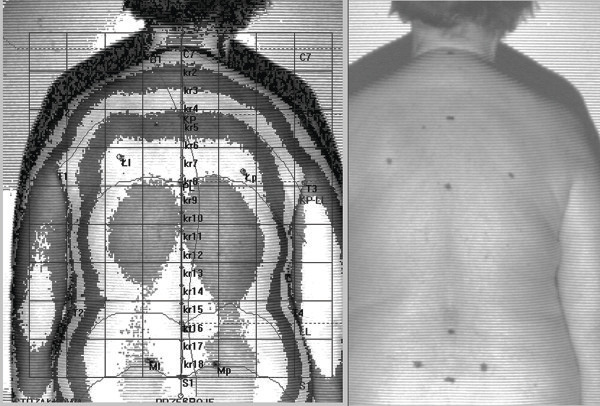
**Photogrammetric survey sample.** Source: own study. The authors obtained the patient’s consent to publish the image.

The following balance assessment parameters were utilised in this study (Table 1).Following the photogram (Figure 1), the following posture assessment parameters were collected during the photogrammetric assessment of the spinal curvature development and used in the analysis:

**Table 1:** The balance assessment parameters utilized in the study

RQSP	Romberg quotient for the length of path of statokinesiogram, which depicts the COP movement during the test in a two-dimensional coordinate system
RQSA	Romberg quotient for COP field marked by the movement of COP in a two-dimensional coordinate system
RQMV	Romberg quotient for the COP means velocity in a two-dimensional coordinate system
RQSPA	Romberg quotient for the COP field quotient to the COP path length
RQSP-L	RQSP quotient for the left platform
RQSA-L: RQSA	Quotient for the left platform
RQMV-L: RQMV	Quotient for the left platform
RQSPA-L: RQSPA	Quotient for the left platform
RQSP-P: RQSP	Quotient for the right platform
RQSA-P: RQSA	Quotient for the right platform
RQMV-P: RQMV	Quotient for the right platform
RQSPA-P: RQSPA	Quotient for the right platform

**Figure 2:**
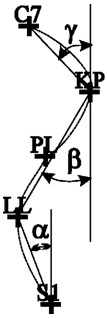
Presents the method of calculating the analyzed parameters.

GKP - Thoracic kyphosis depth, which was calculated in relation to the thoracolumbar junction

GLL - Lumbar lordosis depth, which was calculated in relation to the thoracolumbar junction

KLB - Shoulder line inclination angle

UB - Difference in the depth of the lower angles of the scapula (adjoining the thorax)

UL - Difference in the height of the lower angles of the scapula in the coronal planeUK - The maximum deviation of the line of the spinous processes from the C1–S1 line on the x-axis (Figure 2).

### Statistical analysis

Results were analysed using the Spearman’s rank correlation coefficient test. This nonparametric test was chosen because variables did not exhibit a normal distribution, as determined by the Shapiro-Wilk test. The cut off for statistical significance was set at *p* < 0.05.

## Results

Table 1 describes the balance assessment parameters utilised in the study. The Romberg quotient, which was automatically calculated by the device, expresses the ratio of the parameter values measured in the test with eyes open to the parameter values measured in the test with eyes closed. The Romberg quotient depicts the COP (centre of pressure) movement during the test in a two-dimensional coordinate system, and does this separately for the left and right platform.

To determine the relationship between balance assessment parameters and posture assessment parameters, an analysis using Spearman’s rank correlation coefficient was performed. Tables 2 and 3 present the values of Spearman’s correlation coefficient between pairs of measured parameters. Statistically significant correlations between body posture and balance were found in all cases (p ≤ 0.05). Most of the significant correlations were weak or moderate, and had R-values of 0.20–0.40. However, some slightly stronger positive correlations were found between the quotient for the left platform (RQSA-L) and the lumbar lordosis depth (GLL; R = 0.40), the quotient for the right platform (RQSA-P) and the lumbar lordosis depth (GLL; R = 0.40), and the quotient for the right platform (RQMV-P) and the thoracic kyphosis depth (GKP; R = 0.44). The pair of parameters for which a slightly stronger negative correlation was found was the quotient for the right platform (RQSPA-P) and the lumbar lordosis depth (GLL; R = -0.41).

**Table 2:** Correlation coefficients reflect the Romberg posture assessment

**A pair of variables**	**Spearman rank order correlation**			**A pair of variables**	**Spearman rank order correlation**		
	**R Spearman**	**t(N-2)**	**p**		**R Spearman**	**t(N-2)**	**P**
RQSA-L&GLL	0,40	4,06	0,0001	RQSPA&GKP	0,39	3,93	0,0002
RQSP&GLL	0,23	2,23	0,0286	RQSPA&GLL	-0,33	-3,28	0,0015
RQSA&GKP	-0,34	-3,38	0,0011	RQSP-L &GLL	0,22	2,08	0,0409
RQSA&GLL	0,35	3,46	0,0008	RQSA-L&GKP	-0,34	-3,38	0,0011
RQMV&GLL	0,23	2,23	0,0286				

**Table 3:** Summary of Spearman’s rank correlation test: Romberg coefficients and parameters for assessing the posture photogrammetric evaluation

**A pair of variables**	**Spearman rank order correlation**			**A pair of variables**	**Spearman rank order correlation**		
	**R Spearman**	**t(N-2)**	**p**		**R Spearman**	**t(N-2)**	**P**
RQSP-P&GLL	0,22	2,11	0,0376	RQSA-P&UL	-0,26	-2,54	0,0129
RQMV-L&GLL	0,22	2,08	0,0409	RQSA-P&UB	-0,25	-2,44	0,0169
RQSPA-L&GKP	0,33	3,22	0,0018	RQMV-P&GLL	0,22	2,11	0,0376
RQSPA-L&GLL	-0,39	-3,91	0,0002	RQMV-P&GKP	0,44	4,55	0,0000
RQSPA-L&KLB	0,22	2,09	0,0398	RQSPA-P&GLL	-0,41	-4,14	0,0001
RQSA-P&GKP	-0,37	-3,72	0,0003	RQSPA-P&UL	0,22	2,12	0,0368
RQSA-P&GLL	0,40	4,12	0,0001	RQSPA-P&UB	0,28	2,73	0,0077
				RQSPA-P&UK	0,24	2,33	0,0223

We determined that increased lumbar lordosis depth correlated with an increase in the Romberg quotient value for the length of path, COP field and mean COP velocity, as well as correlating with both the left-side and right-side parameters for length of COP, COP field, and mean COP velocity. On the other hand, lumbar lordosis flattening resulted in a decrease in the Romberg quotient value for the COP path length and COP field, both globally and for left-side and right-side parameters. An increase in the thoracic kyphosis depth resulted in an increase in the Romberg quotients of the COP path length and the COP field in the global assessment and the left-side parameters, as well an increase in the mean COP velocity for the right limb. The Romberg quotient for the COP field decreased globally and for the lateralised parameters (both right- and left-side). An increase in shoulder line asymmetry resulted in an increase in the Romberg quotient parameter for the COP path length and the COP field. We also analysed the height difference between the lower angles of the scapula in the coronal plane. When this parameter increased, the value of the Romberg quotient increased for the COP path length and the COP field for the right-side parameters, while the Romberg quotient value for the COP field decreased for the right limb. A larger difference in the height of the lower angles of the blade in the coronal plane correlated with an increase in the Romberg quotient parameter value for the quotient path length and COP field for the right side. It also correlated with a decrease in the parameter value of the COP field for the right side. The last parameter analysed was the maximal deviation of the line of the spinous processes from the C1–S1 line. An increase in this parameter resulted in a corresponding increase in the quotient for the COP path length and the COP field. Finally, we observed that the results for GLL and GKP were similar in tests performed with both open and closed eyes.

## Discussion

According to Delpech-Wolff’s law, the dynamics and statics of the locomotor system develop continuously during ontogenetic development in humans. The quality and quantity of stimuli during development establish the interrelations between the dynamic and static parts of the locomotor system. Spinal curvatures influence the tension of the paraspinal and postural muscles, which are important components of the balance system [23-26].

The data presented here demonstrate interrelations between the shape of the spinal curvatures and the parameters of static balance quantified in the studied population. The Romberg quotient, which defines the impact of visual control on balance reactions, was used to conduct the analyses [27]. The data presented here, as well as previously published results, indicate that there is an increased dependence on visual contribution during balance reactions [28-30]. We found that this dependence correlated with the degree of spinal curvature in the studied population. On the basis of these results, one can determine whether spinal curvatures play an important role in the assessment of balance reactions in a situation where the visual system is excluded and balance is based on input from proprioceptors and the labyrinth.

Increased lumbar lordosis decreases the dependence of balance reaction quality on visual control through a change in postural muscle tension. Sensitivity to the changes in the COP projection location is caused by an increase in paraspinal or iliopsoas muscle tension [25,31,32]. This phenomenon is only present to a certain extent. A physiological disorder affecting the formation of the spine will always have a negative effect on posture and balance, leading to overloading and postural disorders in response to the changes in muscle tension. On the other hand, deepening of thoracic kyphosis in the studied population had the opposite effect from those previously described. With increased muscle tension, the dependence of balance reaction quality on visual control increased. Movement of the general centre of pressure projection also had an impact, as a new balance situation was created which required a new model of postural control. Changes in muscle tension and spinal curvature require a new or altered model of balance, as the body situation is different than before [31].

Sensitivity of the COP field parameter to visual control increased for a protruding shoulder blade specifically on the protruding side (the right side). An important conclusion of our analyses is that changes in spinal curvature within physiological norms influence tension in postural muscles. This tension models the quality of the response to the change in muscle tension that occurs during movement and free standing. It is a phenomenon that proceeds along a functional track that takes the form of a loop. The output muscle tension conditions the muscle reply to the incoming stimulus, which in turn conditions the formation of the output muscle tension, thus closing the loop [33]. Muscle tension also influences proprioception in the muscle fibres. This was confirmed by analysis of spinal curvature changes in the coronal plane. The direction of the change resulted in an increase in sensitivity of the postural system to correction from the visual system.

The correlation we observed demonstrated that the body posture, the shape of antero-posterior curvatures and the spinal rotation all had an impact on the quality of the response from the balance system. The impact of spinal curvature shape on the proprioceptive sensitivity to changes in muscle tension remains an important issue. Muscle tension is not only the correct reaction to a disruptive factor, but is also necessary to maintain a stable standing position during the static balance test. The effect of muscle tension on the change of muscle excitability and conduction of afferent and efferent information has been demonstrated by numerous studies [19,34].

As the choice of parameters to be analysed was limited, the discussed correlations do not allow for an explicit assessment of the direction of balance parameter change. However, dependences were described on the basis of the literature and the research presented here. To thoroughly evaluate the response of balance systems to spinal curvature, it is necessary to conduct standardised balance tests using muscle tension analysis with the electromyography method. This will allow determination of the precise functional state of specific muscle groups in relation to spinal curvature changes. The fact remains that the function of the locomotor system is to maintain functional balance. The results we observed during the course of this study confirm the ability of certain systems to compensate for irregular spinal curvatures. A thorough analysis of such phenomena is extremely important and useful for the therapeutic reasons. It is well known that functional disorders have a structural basis. Physiotherapy should also contain elements of posture correction, as Hu and Woollacott (1994) have found that balance training designed to improve intersensory interaction could effectively improve balance performance in healthy older adults [35].

## Conclusions

Correlation between the shape of spinal curvature and the static equilibrium of the body depends on visual control. Based on the measurements above, we found that the shape of spinal curvatures influenced postural stability. The improvement of the balance system response depends on corrective information from the visual system and proprioceptive information from the paraspinal muscles. The sensitivity of the balance system to the change of COP location depends on the direction of the change in rotation of the shoulder girdle and the spine. Proper development of spinal curvature in the sagittal plane and the maintenance of symmetry in the frontal and transverse planes are both essential for correct balance control.

### Limitations

Despite its practical value, this study also has some limitations. The authors plan to expand the study group and to include men in future studies. Furthermore, the authors plan to compare how the discussed parameters change in different age groups of women and men. It would be important to analyse whether these variables are age-dependent, to determine which patients should start tailored physiotherapy exercises based on the relationship between posture and balance. It would also be valuable to determine whether a correlation exists between parameters describing the symmetry and stability of posture.

## Competing interests

The authors declare that they have no competing interests.

## Authors’ contribution

JDG contributions to conception and design, acquisition of data, analysis and interpretation of data. JR acquisition of data. MR analysis and interpretation of data, writing. SS has been involved in drafting the manuscript. JP acquisition of data. AT has been involved in drafting the manuscript, revising it critically, and gave final approval of the version to be published. ZT gave final approval of the version to be published. All authors read and approved the final manuscript.

## Pre-publication history

The pre-publication history for this paper can be accessed here:

http://www.biomedcentral.com/1471-2474/15/186/prepub
